# Using a Portable Ventilatory Airway Screening (PVAS) Device to Evaluate the Difference Between Upper Airway Breathing Pressure and Respiratory Flow in Skeletal Class I and Class II Growing Individuals With Retrognathic Mandible

**DOI:** 10.7759/cureus.62898

**Published:** 2024-06-22

**Authors:** Ananya Hazare, Ranjit Kamble, Sunita Shrivastav, Usha Shenoy, Rizwan Gillani

**Affiliations:** 1 Orthodontics, VSPM Dental College and Research Centre, Nagpur, IND; 2 Orthodontics and Dentofacial Orthopaedics, Sharad Pawar Dental College and Hospital, Datta Meghe Institute of Higher Education & Research, Wardha, IND

**Keywords:** non-invasive diagnostic methods, spirometry, skeletal class ii, skeletal class i, pvas device, upper airway obstruction

## Abstract

Background

Upper airway obstruction (UAO) is a significant clinical concern due to its potential to lead to serious health issues, including obstructive sleep apnea (OSA) and cardiovascular diseases. Traditional diagnostic methods, such as spirometry, are often invasive and complex. This study aims to validate a portable ventilatory airway screening (PVAS) device as a non-invasive, cost-effective alternative for measuring upper airway breathing pressure and respiratory flow.

Objectives

To validate the accuracy of the PVAS device in measuring upper airway breathing pressure and respiratory flow by comparing its readings with those obtained from standard spirometry tests.

Methods

This cross-sectional analytical study involved 40 growing individuals aged 10-14 years, divided into two groups based on cephalometric analysis: Skeletal Class I (20 patients) and Skeletal Class II with retrognathic mandible (20 patients). Breathing pressure, volume, and velocity measurements were recorded using both the PVAS device and spirometry, and their accuracy was compared.

Results

The PVAS device showed high concordance with spirometry results, demonstrating significant accuracy in measuring breathing pressure, volume, and velocity. Skeletal Class II individuals exhibited significantly higher breathing pressure and reduced respiratory flow compared to Class I individuals, as measured by the PVAS device.

Conclusion

The PVAS device is a valid and accurate tool for non-invasive measurement of upper airway breathing pressure and respiratory flow. Its ease of use and reliability make it a valuable tool for clinical practice, particularly in the early diagnosis and management of airway obstructions.

## Introduction

Upper airway obstruction (UAO) can lead to serious health issues such as obstructive sleep apnea (OSA), snoring, and significant respiratory difficulties. These conditions severely impact the quality of life and are linked to long-term health risks, including cardiovascular diseases and hypertension [1,2]. Addressing UAO requires comprehensive diagnostic approaches, typically involving invasive methods, such as spirometry, which may cause discomfort and are complex to administer [3].

Skeletal malocclusions, particularly Class I and Class II, significantly affect the upper airway's structure and functionality [4,5]. Skeletal Class I malocclusion involves normal alignment between the upper and lower jaws. In contrast, Class II malocclusion involves a retrusive lower jaw, predisposing individuals to airway obstruction by causing posterior displacement of the tongue and soft palate [6,7]. Understanding these anatomical relationships is crucial for accurate diagnosis and effective management of UAO [8,9].

Portable Ventilatory Airway Screening (PVAS) Device

A PVAS device presents a non-invasive, cost-effective tool used alongside dental chairs in clinics, measuring airway obstruction induced by skeletal discrepancies. This device offers a practical alternative to conventional diagnostic methods such as spirometry, rhinomanometry, and polysomnography [10,11]. The PVAS device enhances diagnostic accuracy, patient comfort, and early intervention capabilities, providing substantial benefits for patients, healthcare providers, and researchers [12,13].

## Materials and methods

Study Design

This cross-sectional analytical study aimed to measure upper airway breathing pressure and respiratory flow in growing individuals with the help of a PVAS device. The study was conducted in the Department of Orthodontics and Dentofacial Orthopaedics, Sharad Pawar Dental College, Wardha.

Population

The study involved 40 growing individuals aged 10-14 years, divided into two groups based on their skeletal class and mandibular position:

Group 1: Skeletal Class I (20 patients): Individuals with normal alignment between the upper and lower jaws.

Group 2: Skeletal Class II with retrognathic mandible (20 patients): Individuals with a retrusive lower jaw, predisposing them to airway obstruction.

Inclusion Criteria

Participants were included in the study if they willingly participated in the research after informed consent. They had no history of prior orthodontic treatment. Patients had either skeletal Class I or II malocclusion and were between the ages of 10 and 14 years.

Exclusion Criteria

Participants were excluded from the study if they were diagnosed with any syndromes. Exhibited facial asymmetry, nasal deformity, or a deviated nasal septum. If the patients had a cough or cold at the time of the evaluation they were excluded from the study. Patients having a history of COVID-19 were excluded. Patients having a history of asthma or any other breathing disorders were also excluded.

Data Collection

**Figure 1:**
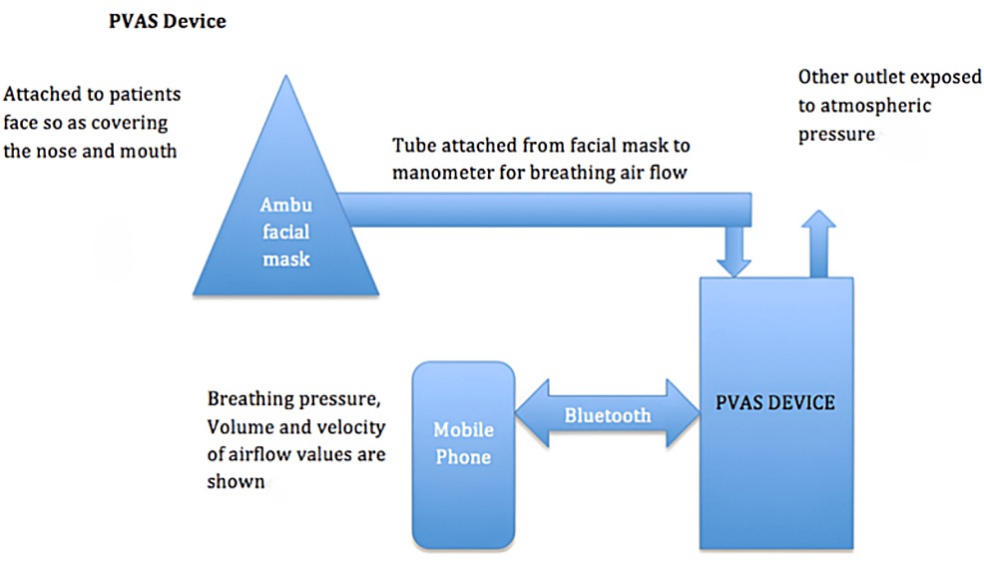
Schematic representation of the portable ventilatory airway screening (PVAS) device

The following data were collected for each participant: Breathing pressure, volume, and velocity were measured using the PVAS device (testo 510i). The schematic representation of the PVAS device is presented in Figure 1.

The skeletal class and retroposition of the mandible were determined through cephalometric analysis. Demographic information such as age and gender was also recorded.

Procedure

Patient Preparation

Participants were instructed to avoid strenuous activity and caffeine consumption for at least 12 hours before the measurements. Participants were asked to refrain from eating or drinking for at least two hours before the measurements. Participants were prepared with oxymetazoline hydrochloride nasal spray and warm saline to clear nasal passages before measurement.

PVAS Measurement

**Figure 2:**
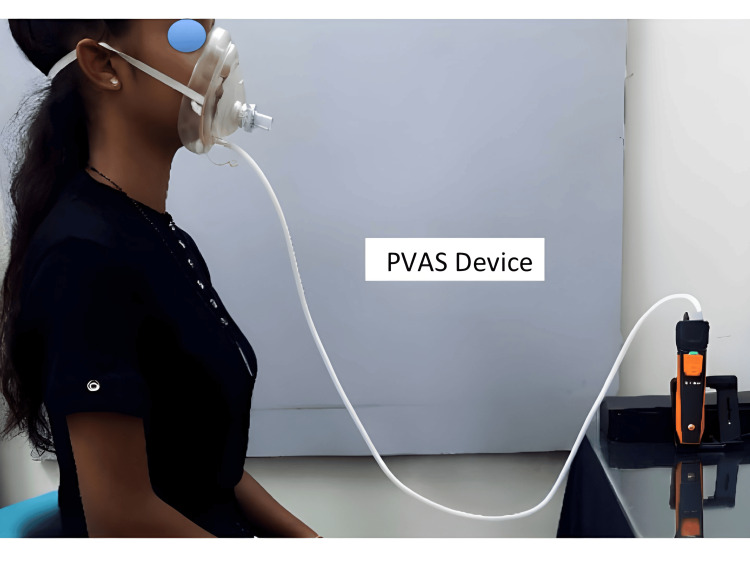
Patient's readings taken with the portable ventilatory airway screening (PVAS) device

The PVAS device was calibrated according to the manufacturer's instructions. Participants were seated comfortably with their head and neck in a neutral position. The PVAS mouthpiece was placed in the participant's mouth and secured. Participants were instructed to breathe normally through the mouthpiece for a period of five minutes. The PVAS device recorded the average breathing pressure, volume, and velocity data during this period (Figure 2).

Cephalometric Analysis

**Figure 3:**
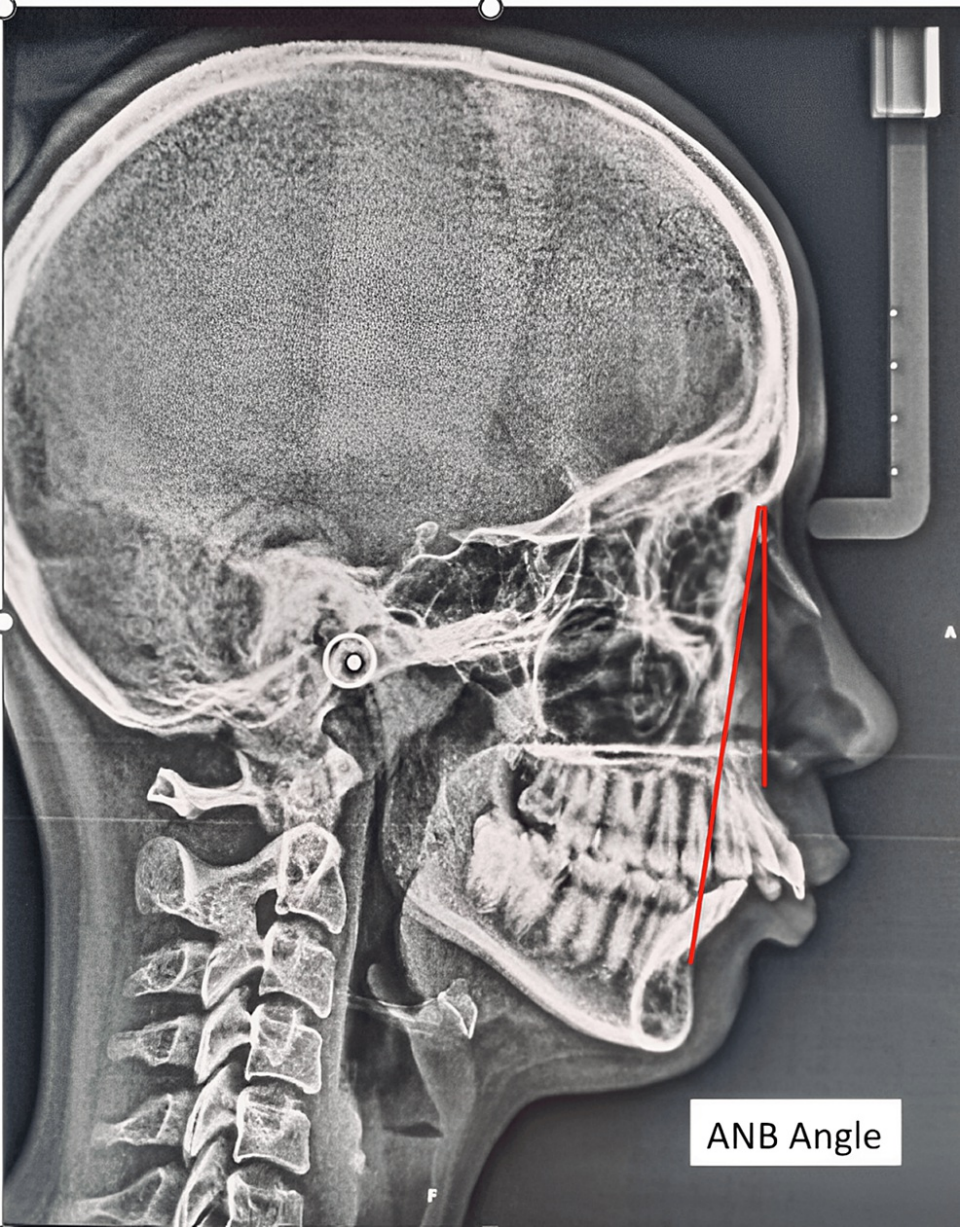
ANB angle

**Figure 4:**
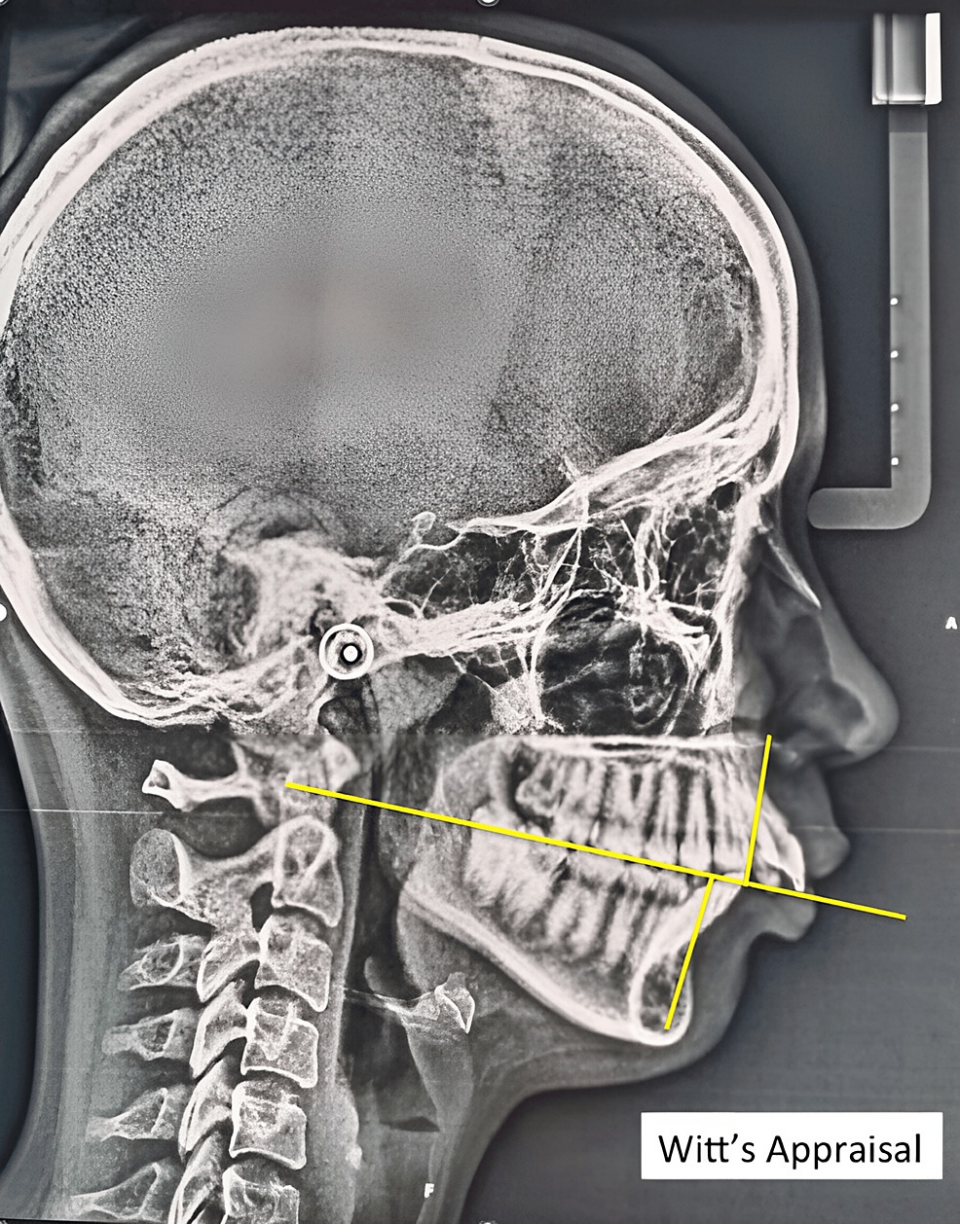
Witt's appraisal

**Figure 5:**
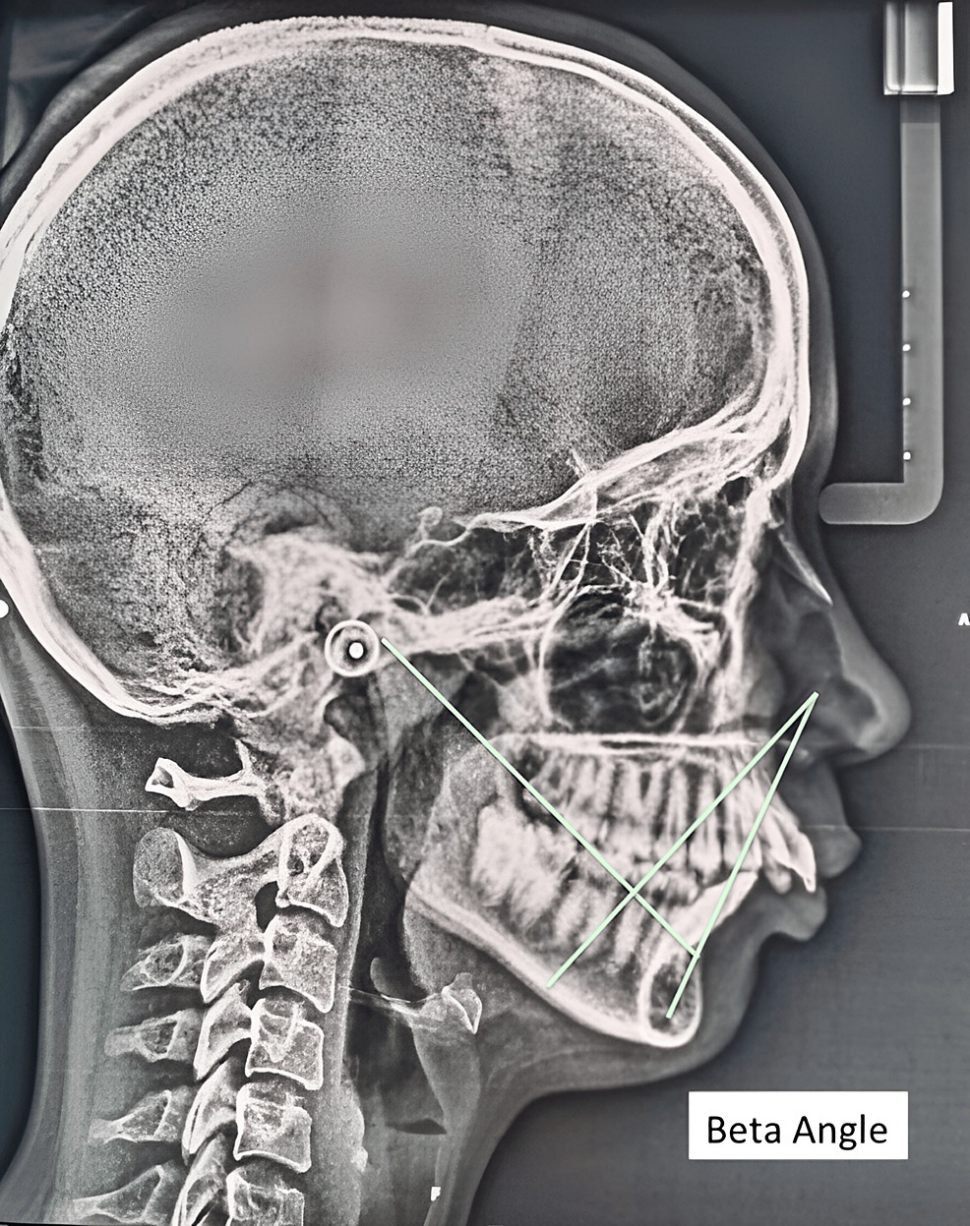
Beta angle

**Figure 6:**
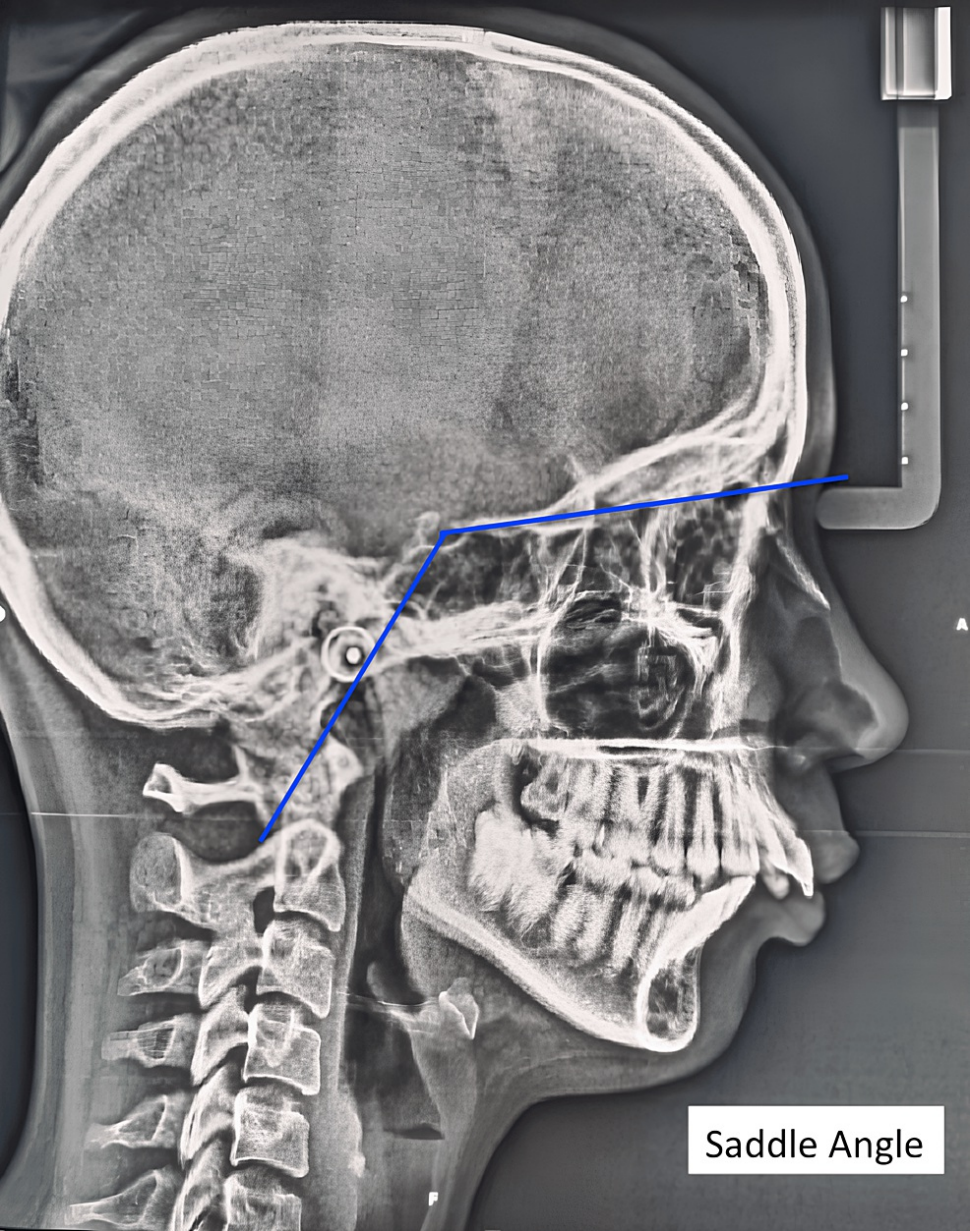
Saddle angle

Lateral cephalometric radiographs were taken for all participants. The radiographs were analyzed using specialized software to determine the skeletal class and the presence of mandibular retroposition. The following cephalometric angles were measured: ANB angle (Figure 3), Witt's appraisal (Figure 4), beta angle (Figure 5), and saddle angle (Figure 6).

Statistical Analysis

Data were analyzed using Statistical Product and Service Solutions (SPSS, version 21; IBM SPSS Statistics for Windows, Armonk, NY). Intragroup comparisons were conducted using paired t-tests. Intergroup comparisons were made using one-way ANOVA. Statistical significance was set at p < 0.05.

Justification of Methods

The PVAS device was chosen for its non-invasiveness, portability, and ease of use. The cephalometric analysis provided a reliable method for assessing skeletal class and mandibular position. The chosen statistical tests were appropriate for the study design and data distribution.

## Results

Analyzing Table 1, the mean exhalation volume for skeletal Class I individuals was 2.3778, significantly higher than the inhalation volume of 1.6111 (p < 0.001). This finding indicates increased expiratory capacity in this group.

**Table 1:** Comparison of volume indicates a significantly higher exhalation volume than the inhalation volume (p < 0.001)

Group	N	Mean	Std. Deviation	Std. Error Mean	t-value	P-value
Volume						
Inhalation	20	1.6111	0.31002	0.10334	-4.28	<0.001
Exhalation	20	2.3778	0.43811	0.14604		

Similarly, Table 2 demonstrates a significant difference in velocity, with exhalation velocity (25.8656) exceeding inhalation velocity (16.4956) in Class I individuals (p < 0.001). This suggests faster air expulsion during exhalation.

**Table 2:** Velocity comparison shows a higher exhalation velocity (p < 0.001)

Group	N	Mean	Std. Deviation	Std. Error Mean	t-value	P-value
Velocity						
Inhalation	20	16.4956	1.54179	0.51393	-7.831	<0.001
Exhalation	20	25.8656	3.24169	1.08056		

Table 3 reveals a significantly lower mean exhalation pressure (-34.5256) compared to inhalation pressure (15.9000) for Class I individuals (p < 0.001). This signifies reduced pressure generation during exhalation, potentially indicating a less obstructed airway.

**Table 3:** Pressure comparison indicates a significantly lower exhalation pressure (p < 0.001)

Group	N	Mean	Std. Deviation	Std. Error Mean	t-value	P-value
Pressure						
Inhalation	20	15.9000	0.76974	0.25658	67.57	<0.001
Exhalation	20	-34.5256	2.10234	0.70078		

Analyzing Table 4, the mean exhalation volume for skeletal Class II individuals was 1.8778, significantly higher than the inhalation volume of 1.1667 (p < 0.001). This finding aligns with the observation for Class I individuals, indicating increased expiratory capacity.

**Table 4:** Exhalation volume is significantly higher than the inhalation volume (p < 0.001)

Group	N	Mean	Std. Deviation	Std. Error Mean	t-value	P-value
Volume						
Inhalation	20	1.1667	0.32787	0.10929	-4.12	<0.001
Exhalation	20	1.8778	0.39930	0.13310		

Table 5 demonstrates a significant difference in velocity, with exhalation velocity (22.5256) exceeding inhalation velocity (13.0911) in Class II individuals (p < 0.001). This suggests faster air expulsion during exhalation, similar to Class I individuals. 

**Table 5:** Exhalation velocity is notably higher (p < 0.001)

Group	N	Mean	Std. Deviation	Std. Error Mean	t-value	P-value
Velocity						
Inhalation	20	13.0911	1.80690	0.60230	-8.06	<0.001
Exhalation	20	22.5256	3.00993	1.00331		

Table 6 reveals a significantly lower mean exhalation pressure (-43.9078) compared to inhalation pressure (18.5667) for Class II individuals (p < 0.001). This finding aligns with the observation for Class I individuals, signifying reduced pressure generation during exhalation, potentially indicating a less obstructed airway.

**Table 6:** Exhalation pressure is significantly lower than the inhalation pressure (p < 0.001)

Group	N	Mean	Std. Deviation	Std. Error Mean	t-value	P-value
Pressure						
Inhalation	20	18.5667	1.30767	0.43589	54.76	<0.001
Exhalation	20	-43.9078	3.16278	1.05426		

Summary of Findings

The study confirms that individuals with skeletal Class II malocclusion exhibit significantly higher breathing pressure and reduced respiratory flow compared to Class I individuals. These findings highlight the influence of mandibular retrognathia on upper airway dynamics. The PVAS device proved effective in capturing these differences, demonstrating its potential as a valuable tool in the diagnosis and management of airway issues.

## Discussion

The current study adds to the growing body of evidence highlighting the significant impact of skeletal malocclusions, particularly Class II, on upper airway dynamics in growing individuals. The observed differences in breathing pressure, respiratory flow, and velocity between Class I and Class II individuals underscore the importance of recognizing the influence of mandibular retrognathia on airway resistance. Delving deeper into the findings, we observed the following.

Higher Exhalation Volumes and Velocities in Class II Individuals

The study's findings align with previous research by Kannan et al. [14] and Sinha et al. [15], who demonstrated increased airway resistance in individuals with retrognathic mandibles. This suggests that the retrusive lower jaw position in Class II individuals leads to a narrowing of the airway, making it more challenging to expel air during exhalation. This phenomenon can have implications for various respiratory functions, including speech and exercise performance and overall growth of the individual.

Lower Mean Inhalation Volumes in Class I Individuals

Florez et al. [16] reported lower mean inhalation volumes in Class III individuals compared to their Class I counterparts. This can be explained by the influence of facial skeletal structure on lung capacity. Individuals with Class I malocclusion have a normal alignment between the upper and lower jaws, resulting in a more optimal airway configuration that allows for greater lung expansion during inhalation. This finding highlights the potential impact of skeletal discrepancies on overall respiratory health and efficiency.

The Complex Interplay Between Skeletal Development and Respiratory Function

The observed differences in breathing parameters between Class I and Class II individuals underscore the complex interplay between skeletal development and respiratory function. Understanding the underlying mechanisms responsible for these differences is crucial for developing effective treatment strategies for individuals with UAOs. This knowledge can inform orthodontic and surgical interventions aimed at improving both airway patency and overall respiratory function.

The study emphasizes the importance of early detection and intervention for individuals with UAOs. Early identification allows for prompt implementation of appropriate measures, potentially mitigating the long-term consequences of these conditions. This can include non-invasive interventions such as myofunctional therapy or more invasive approaches such as orthodontic treatment or surgery, depending on the severity of the obstruction. Advanced diagnostic tools such as the PVAS device, along with precise imaging techniques such as those employed by one study [17], offer increased accuracy in identifying UAOs. This allows clinicians to make informed decisions about treatment plans and monitor the effectiveness of interventions over time. Accurate diagnosis is crucial for ensuring optimal patient outcomes and avoiding unnecessary interventions.

Longitudinal studies for personalized treatment approaches. A study done by Ucar et al. [18] provides valuable insights into the evolution of airway parameters throughout growth in different growth patterns. Understanding how airway dimensions and resistance change over time allows for customized treatment approaches that address the specific needs of each individual. This personalized approach can maximize the effectiveness of treatment and improve long-term outcomes.

Embracing a multidisciplinary approach through a collaboration between orthodontists, surgeons, and respiratory specialists is necessary to manage cases with mandibular retrognathism and obstructed airways. The study highlights the importance of a multidisciplinary approach involving orthodontists, surgeons, and respiratory specialists in managing UAOs. Orthodontic interventions, as demonstrated by Ucar et al. [19], can significantly improve airway volumes in Class II individuals by correcting the underlying skeletal malocclusion. In cases where orthodontic treatment alone is insufficient, surgical interventions may be necessary to address more severe airway obstruction. Respiratory specialists play a crucial role in evaluating respiratory function, monitoring treatment effectiveness, and managing any associated respiratory complications. This collaborative approach ensures comprehensive care and optimal outcomes for patients with UAOs.

Looking toward the future, researchers are poised to delve deeper into the intricate world of upper airway dynamics and skeletal malocclusions, paving the way for groundbreaking advancements in diagnosis, treatment, and prevention.

Need for larger-scale studies for robust evidence: Expanding the scope of research through larger-scale studies will provide invaluable insights into the effectiveness of the PVAS device and the intricate relationship between malocclusions and upper airway function. This robust evidence will empower the development of evidence-based treatment guidelines, ensuring optimal care for individuals affected by these conditions. Longitudinal studies for long-term outcome evaluation should be done. By meticulously tracking changes in airway parameters over time through longitudinal studies, researchers can gain a deeper understanding of the long-term outcomes of various treatment interventions. This knowledge will guide the continuous improvement of patient care, ensuring that treatment approaches are not only effective but also sustainable over the long term. Investigating the role of genetics and environment is an important factor that should be researched further. Unraveling the complex interplay between genetics, environment, and skeletal development in relation to upper airway function is crucial. This line of inquiry will illuminate the factors influencing airway development, paving the way for the development of preventative strategies to combat UAOs before they arise. The future holds immense potential for the development of innovative diagnostic tools and treatment approaches for UAOs. This includes exploring new technologies for airway assessment, enabling more accurate and efficient diagnosis. Additionally, the development of minimally invasive and effective treatment options will revolutionize patient care, minimizing discomfort and maximizing positive outcomes. By pursuing these avenues of research, the medical community can make significant strides in understanding, diagnosing, and treating UAOs, ultimately improving the quality of life for individuals affected by these conditions. 

This study significantly contributes to our understanding of the impact of skeletal malocclusions on upper airway dynamics in growing individuals. The findings highlight the importance of early detection, multidisciplinary treatment approaches, and ongoing research in this critical area of healthcare. By continuing to explore the complexities of UAOs and develop innovative solutions, we can improve the lives of individuals affected by these conditions.

## Conclusions

This study conclusively demonstrates the detrimental effects of mandibular retrognathia on respiratory function in growing individuals. The misalignment constricts the upper airway, leading to increased breathing pressure, decreased respiratory flow, and potential long-term health complications. Early detection and targeted interventions are crucial in mitigating these effects, and the PVAS device emerges as a valuable tool in this endeavor.

The study successfully validates the PVAS device as a reliable and accurate alternative to traditional spirometry. Its non-invasive nature, user-friendliness, and ability to provide comprehensive data on breathing pressure and flow empower healthcare professionals to personalize treatment plans and optimize respiratory health outcomes for patients with mandibular retrognathia. The seamless integration of the PVAS device into routine clinical practice represents a significant advancement in managing this condition and ensuring optimal respiratory function for this patient population.

## References

[1] Ponnada SR, Ganugapanta VR, Perumalla KK, Naqeed MA, Harini T, Mandaloju SP (2020). Airway analysis in skeletal class I and class II subjects with different growth patterns: a 2D cephalometric study. J Pharm Bioallied Sci.

[2] Becking BE, Verweij JP, Kalf-Scholte SM, Valkenburg C, Bakker EWP, van Merkesteyn JPR. (2017). Impact of adenotonsillectomy on the dentofacial development of obstructed children: a systematic review and meta-analysis. Eur J Orthod.

[3] Rodrigues J, Evangelopoulos E, Anagnostopoulos I (2024). Impact of class II and class III skeletal malocclusion on pharyngeal airway dimensions: a systematic literature review and meta-analysis. Heliyon.

[4] Singh D (2023). A new treatment for chronic obstructive pulmonary disease: ensifentrine moves closer. Am J Respir Crit Care Med.

[5] Paul D, Varma S, Ajith VV (2015). Airway in Class I and Class II skeletal pattern: a computed tomography study. Contemp Clin Dent.

[6] Silva NN, Lacerda RH, Silva AW, Ramos TB (2015). Assessment of upper airways measurements in patients with mandibular skeletal Class II malocclusion. Dental Press J Orthod.

[7] Perossi L, Holtz M, Santos DO, Perossi J, Souza HC, Salgado Junior W, Gastaldi AC (2022). Increased airway resistance can be related to the decrease in the functional capacity in obese women. PLoS One.

[8] Lin J, Bellinger R, Shedd A (2023). Point-of-care ultrasound in airway evaluation and management: a comprehensive review. 2023.

[9] Indriksone I, Jakobsone G (2014). The upper airway dimensions in different sagittal craniofacial patterns: a systematic review. Stomatologija.

[10] Pop SI, Procopciuc A, Arsintescu B (2024). Three-dimensional assessment of upper airway volume and morphology in patients with different sagittal skeletal patterns. Diagnostics.

[11] Abbasi A, Gupta SS, Sabharwal N, Meghrajani V, Sharma S, Kamholz S, Kupfer Y (2021). A comprehensive review of obstructive sleep apnea. Sleep Sci.

[12] Chianchitlert A, Luppanapornlarp S, Saenghirunvattana B, Sirisoontorn I (2022). A comparative assessment of the upper pharyngeal airway dimensions among different anteroposterior skeletal patterns in 7-14-year-old children: a cephalometric study. Children (Basel).

[13] He H, Zhang S, Xu J (2023). Impact of occlusal reconstruction positions on airway dimensions in patients with edentulism and long centric occlusion. BMC Oral Health.

[14] Kannan A, Sathyanarayana HP, Padmanabhan S (2017). Effect of functional appliances on the airway dimensions in patients with skeletal class II malocclusion: a systematic review. J Orthod Sci.

[15] Sinha SP, Nayak KU, Soans CR, Murali PS, Shetty A, Ravi MS (2018). Assessment of mandibular retrognathism and maxillary prognathism as contributory factors for skeletal Class II malocclusion: a cephalometric study. Int J Oral Health Sci.

[16] Florez BM, Tagawa DT, Inoue DP, Yamashita HK, Aidar LAA, Dominguez GC (2023). Associations between skeletal discrepancies, breathing pattern, and upper airway obstruction in Class III malocclusions. Int J Pediatr Otorhinolaryngol.

[17] Major MP, Witmans M, El-Hakim H (2014). Major PW, Flores-Mir C. Agreement between cone-beam computed tomography and nasoendoscopy evaluations of adenoid hypertrophy. Am J Orthod Dentofacial Orthop.

[18] Ucar FI, Uysal T (2011). Orofacial airway dimensions in subjects with Class I malocclusion and different growth patterns. Angle Orthod.

[19] Pliska BT, Tam IT, Lowe AA, Madson AM, Almeida FR (2016). Effect of orthodontic treatment on the upper airway volume in adults. Am J Orthod Dentofacial Orthop.

